# 

**Published:** 1916-02-19

**Authors:** 


### CONTENTS.

PAGE

**Infectious Disease and its Notification** ... 441

**The Treatment of Disabled Soldiers** ... ... 442

**Hospital and Institutional News** ... ... ... 443

Long Life in Personal Service—The Medical Profession and the War—The Central Medical War Committee—Expressions of Dissatisfaction—Meetings and Deputations—Security of Tenure for Medical Officers of Health—Secretaryship of the Norfolk and Norwich Hospital—A Strike at Bradford—The Royal Infirmary, Edinburgh—The Nursing Staff and a Test of Efficiency—A Rapid Reorganisation—An Honorarium for the Honorary Staff—The Cut and Colour of Hospital Clothes—More Infirmary Beds for Soldiers—"Shell-Shock" and Suicide—The Drug Habit Among Soldiers—The Sale of Morphine to Army Officers—Wanton Procedure of Alexandra Day Managers—A Poor-Law Medical Officer's Protest—The "Religious Rights" of the Wounded—Illustrators and Writers on the New Hospital *Gazettes*—The Poor-Law Mattress—The Overworked Health Visitor—The Royal Sanitary Institute—No Economy in Health—A Point for Hospital Surgeons and Managers—This Week's Drug Market.

**The Soldier's Heart** ... ... ... ... ... ... 449

A Medical Aspect of War Wastage.

**The Law in Institutional Practice** ... ... 450

An Important Pronouncement.

**Florence Nightingale, O.M.** ... ... ... ... 451

The Memorial Service at St. Paul's Cathedral.

PAGE

**Hospitals and the Poets** ... ... ... ... ... 453

IV. Crabbe, Akenside, and Armstrong.

**Margarine versus Butter** ... ... ... ... 454

**Hospital Economics in War-Time** ... ... 455

St. Bartholomew's Hospital Action.

**Food Economy in Institutions** ... ... ... 456

How to Prepare a Menu.

**Memorial to Miss Katharine Monk** ... ... 456

**The Matron's and Sister's Department** ... 457

A Matron's Problem.
Are Assistant Matrons Unbusinesslike?
Voluntary Hospital Workers at their Best.
The Soldier's Question.
The Burdens of a Principal Matron.
Dr. Chadburn's Sink Steriliser.
Sisters in Children's Hospitals.

**The Status and Teaching of Massage** ... ... 459

The Incorporated Society of Trained Masseuses.

**Passing Events and Topics** ... ... ... ... 460

**Textile Supplies for Hospitals** ... ... ... 461

V. The Methods of Manufacture.

**The Editor's Letter-Box** ... ... ... ... 463

Some Interesting Problems.

**Memorial to Miss Florence Nightingale, O.M.** 464

Form of Service.

**Some Hospital Chapels** ... ... ... ... 464

Royal Mineral Water Hospital, Bath.

**Medical Appointments** ... ... ... ... ... iv

**Institutional Needs** ... ... ... ... ... iv

**The Institutional Worker** ... ... (Suppl.) 1

